# Dataset of (±)-NBI-74330 (CXCR3 antagonist) influence on chemokines under neuropathic pain

**DOI:** 10.1016/j.dib.2018.10.091

**Published:** 2018-10-26

**Authors:** Anna Piotrowska, Ewelina Rojewska, Katarzyna Pawlik, Grzegorz Kreiner, Agata Ciechanowska, Wioletta Makuch, Joanna Mika

**Affiliations:** a Institute of Pharmacology, Polish Academy of Sciences, Department of Pain Pharmacology, Smetna Street 12, 31-343 Krakow, Poland; b Institute of Pharmacology, Polish Academy of Sciences, Department of Brain Biochemistry, Smetna Street 12, 31-343 Krakow, Poland

## Abstract

Our data give evidence that CXCR3 ligands exhibit pronociceptive properties and play an important role in the initiation, development and maintenance of neuropathic pain. Moreover, intrathecal administration of each CXCR3 ligand induced hypersensitivity reactions in naive mice and of its neutralizing antibodies diminished neuropathic pain syndrome in CCI-exposed mice. Furthermore, our results indicate that selective CXCR3 antagonist (±)-NBI-74330 reduced the neuropathic pain-related behaviour and also enhanced morphine analgesic potency in CCI-exposed rats. Interestingly, our data show that (±)-NBI-74330 administration diminished the spinal IBA1 and, in parallel, downregulated CXCL4, CXCL9 and CXCL10. In addition, CXCR3 antagonist increased the spinal GFAP, what correlates with upregulation of CXCR3 and CXCL11. Moreover, in DRG (±)-NBI-74330 did not change IBA1 and GFAP positive cells activation, however downregulated also CXCL9. CXCR3 and CXCL10 were co-localized predominantly with neuronal marker in the spinal cord. Summing up, chronic (±)-NBI-74330 intrathecal injection promotes beneficial analgesic effects in rat neuropathic pain model, as described in details in “Pharmacological blockade of CXCR3 by (±)-NBI-74330 reduces neuropathic pain and enhances opioid effectiveness - evidence from *in vivo* and *in vitro* studies” (Piotrowska et al., 2018).

**Specifications table**

**Table:** 

Subject area	Neuroscience
Specific subject area	Neuropathic pain
Type of data	Figures, Table
How data was acquired	1. BEHAVIORAL ANALYSIS:
	• Tactile stimulus - von Frey test (dynamic Plantar Aesthesiometer, Ugo Basile, and classical von Frey filaments, Stoelting) • Thermal stimulus - cold plate test (Hot/Cold Plate Analgesia Meter, Columbus Instruments)
	2. BIOCHEMICAL ANALYSIS:
	• Protein analysis -Western blot (Western blotting system Bio-Rad); immunohistochemistry (sample preparation – Leica Paraffin Station and rotary microtome; sample processing – standard protocol with Alexa fluorescent secondary antibodies; visualization – Nikon Eclipse 50i microscope)
Data format	Schemes, Figures, Table
Experimental factors	The chemicals used were obtained from the following sources:
	• (±)-NBI-74330 (Tocris, Warsaw, Poland) was reconstituted at 10 mg/mL in DMSO to prepare the stock solutions, which then was diluted to the doses used in the experiment (NBI, 10 μg/5 μL, *i.t*.) • Morphine TEVA, Kutno, Poland was weighed and dissolved in water for injection to the dose used in the study (M; 2.5 μg/5 μL, *i.t*.) • CXCL4, CXCL9, CXCL10, CXCL11, CCL21 (R&D Systems, USA) were reconstituted at 0.1 mg/mL in water for injection to prepare the stock solutions, which then was diluted to the doses desired in the experiment (2, 200, and 400 ng/5 μl, *i.t*.) • CXCL4, CXCL9, CXCL10, CXCL11, CCL21 neutralizing antibodies (R&D Systems, USA) were reconstituted at 2 mg/mL in water for injection to prepare the stock solutions, which then was diluted to the doses used in the experiment (4 and 8 µg/5 μL, *i.t*.)
Experimental features	The experiments were carried out according to IASP and NIH rules for the care and use of laboratory animals. The study protocol was approved by the II Local Bioethics Committee branch of the National Ethics Committee for Experiments on Animals based at the Institute of Pharmacology, Polish Academy of Sciences (Krakow, Poland), permission number: 1277/2015 and 262/2017. Neuropathic pain model - Chronic Constriction Injury (CCI model) of the sciatic nerve was performed according to Bennett and Xie (1988). Drugs intrathecal administration: rats were prepared for the *i.t.* injection by the insertion of catheter, according to Yaksh and Rudy, (1976); mice *i.t.* injection procedure was performed according to Hylden and Wilcox (1980). The influence of single intrathecal administration of CXCR3 ligands and its neutralizing antibodies was performed in naïve and in CCI-exposed mice, respectively. The analgesic effects of (±)-NBI-74330 and morphine were measured in naïve an CCI-exposed rats. The biochemical study was performed to examine the (±)-NBI-74330 influence on CCI-induced changes on IBA1, GFAP and CXCR3 ligands protein levels and the time course of changes in proteins levels of the chemokines in the spinal cord and DRG in rats. The spinal localization of CXCR3 and CXCL10 determined at day 7 after CCI in rats.
Data source location	Krakow, Poland
Data accessibility	Data within this article
Related research article	Piotrowska A., Rojewska E., Pawlik K., Kreiner G., Ciechanowska A., Makuch W., Zychowska M., Mika J. Pharmacological blockade of CXCR3 by (±)-NBI-74330 reduces neuropathic pain and enhances opioid effectiveness - evidence from in vivo and in vitro studies. Biochim Biophys Acta Mol Basis Dis. 1864(10):3418–3437 [1]

**Value of the data**

• These data provide that CXCR3 ligands (CXCL4, CXCL9, CXCL10, CXCL11, CCL21) are important in the initiation, development and/or maintenance of neuropathic pain.
• These data provide characterization of pronociceptive properties of CXCR3 ligands and cellular source of these factors.
• The data support the development of research on neutralizing antibodies of CXCR3 ligands (CXCL9, CXCL10 and CCL21) in diminishing tactile and thermal hypersensitivity.
• The data would be valuable for further studies on the effect of (±)NBI-74330 on glial cells modulations and the release of CXCR3 ligands and other pronociceptive factors.
• These data could give a basis for further experiments on revealing the underlying mechanism of action of the (±)NBI-74330 in reducing the symptoms of neuropathic pain and enhancing the effectiveness of morphine.

## 1 Data

**Scheme 1:**
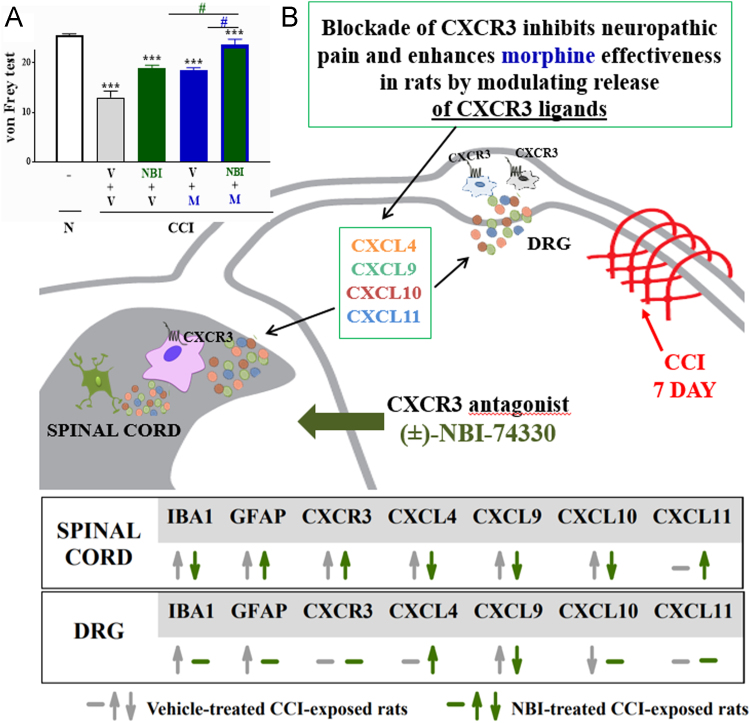
Graphical abstract showing the influence of repeated administration of CXCR3 antagonist (±)-NBI-74330 on A. pain-related behaviours & morphine effectiveness and B. IBA1, GFAP, CXCR3, CXCL4, CXCL9, CXCL10, CXCL11 protein levels in the spinal cord and DRG 7 days after CCI in rats.

Chronic intrathecal (±)-NBI-74330 administration significantly attenuated pain-related behaviour and enhanced the analgesic properties of morphine (Scheme 1A). The Western blot analysis provided evidence that CCI-induced the upregulation of microglial and astroglial markers, CXCR3 ligands (CXCL4, CXCL9, CXCL10) protein levels in the ipsilateral lumbar spinal cord and IBA1 and GFAP positive cells activation, and CXCL9 in the DRG. Interestingly, (±)-NBI-74330 significantly diminished microglial and increased astrocytes activation and downregulated the spinal expression of CXCL4, CXCL9 and CXCL10 in the spinal cord 7 days after CCI. In the DRG, (±)-NBI-74330 decreased CCI-upregulated CXCL9 (Scheme 1B).

## 2 Experimental design, materials and methods

### 2.1 Animals

In our experiments we used male Wistar rats and Albino Swiss mice from Charles River (Sulzfeld, Germany)

### 2.2 Neuropathic pain model

**Scheme 2:**
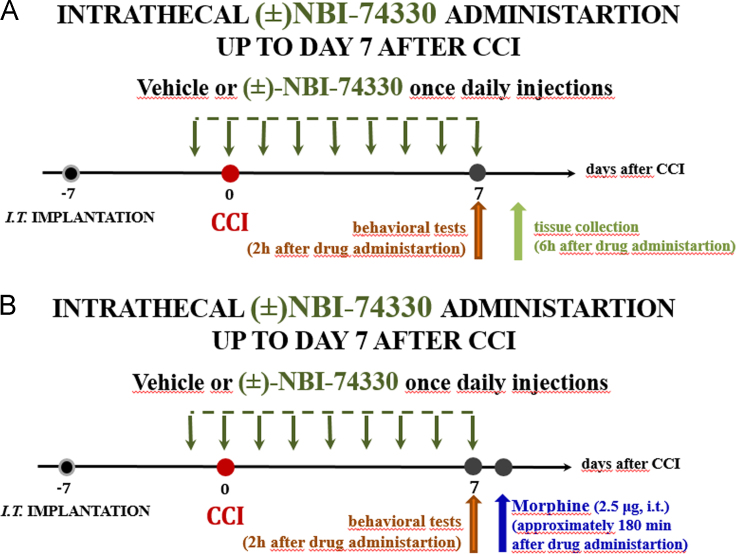
A. The way of (±)-NBI-74330 administration for behavioural and Western blot analysis on day 7 after chronic constriction injury (CCI) to the sciatic nerve. B. The way of co-administration of (±)-NBI-74330 and morphine for behavioural analysis on day 7 after CCI to the sciatic nerve .

Bennett model, recognized widely as CCI model due to its unilateral Chronic Constriction Injury to the right sciatic nerve in rats and mice (Scheme 2) [1], [2], [3], [4].

### 2.3 Study design in rat neuropathic pain model

#### 2.3.1 First stage of behavioural studies

The chronic administration of (±)-NBI-74330 (NBI, 10 μg/5 μl) or vehicle (V) were provided once a day 16 h (day 1) and 1 h (day 0) before CCI and for 7 next days (like maraviroc [4]). On the 7th day post-CCI behavioural tests were conducted 120 min (von Frey test) and 125 min (cold plate test) after NBI or V administration and then 6 h (for Western blot analysis) after the last NBI or V injection the tissue was collected (Scheme 2A) [1].

#### 2.3.2 Second stage of behavioural studies

The co-administration of chronic (±)-NBI-74330 with opioids were performed. The group of repeatedly NBI- or V-treated rats received on the 7th day after CCI (approximately 180 min after NBI or V injection) a single injection of morphine (2.5 μg/5 μl), and then, the von Frey and cold plate tests were conducted. *Abbreviations*: CCI, chronic constriction injury; DRG, dorsal root ganglia; *i.t*., intrathecal; NBI, (±)NBI-74330; V, vehicle; M, morphine (Scheme 2B) [1].

### 2.4 Study design in naïve mice and in mouse neuropathic pain model

#### 2.4.1 Third stage of behavioural studies

The influence of single intrathecal administration of CXCL4, CXCL9, CXCL10, CXCL11, CCL21 on nociceptive transmission in naive mice.

#### 2.4.2 Fourth stage of behavioural studies

The influence of single intrathecal administration of CXCL9, CXCL11, CCL21 neutralizing antibodies on pain-related behaviours in CCI-exposed in mice.

## References

[1] Piotrowska A., Rojewska E., Pawlik K., Kreiner G., Ciechanowska A., Makuch W., Zychowska M., Mika J. (2018). Pharmacological blockade of CXCR3 by (±)-NBI-74330 reduces neuropathic pain and enhances opioid effectiveness - evidence from in vivo and in vitro studies. Biochim. Biophys. Acta Mol. Basis Dis..

[2] Bennett G.J., Xie Y.K. (1988). A peripheral mononeuropathy in rat that produces disorders of pain sensation like those seen in man. Pain.

[3] Yaksh T.L., Rudy T.A. (1976). Chronic catheterization of the spinal subarachnoid space. Physiol. Behav..

[4] Kwiatkowski K., Piotrowska A., Rojewska E. (2016). Beneficial properties of maraviroc on neuropathic pain development and opioid effectiveness in rats. Prog. Neuropsychopharmacol. Biol. Psychiatry.

