# Supplementary material for: Dataset of (±)-NBI-74330 (CXCR3 antagonist) influence on chemokines under neuropathic pain
Source: Data Brief. 2018 Oct 26;21:1145–50. doi: 10.1016/j.dib.2018.10.091 (PMC6231032; doi:10.1016/j.dib.2018.10.091)
Supplement: Supplementary file 1 — Supplementary material [file mmc1.doc]

**CONFLICT OF INTEREST STATEMENT**

The authors (Anna Piotrowska, Ewelina Rojewska, Katarzyna Pawlik, Grzegorz Kreiner, Agata Ciechanowska, Wioletta Makuch, Joanna Mika) of the manuscript “Dataset of (±)-NBI-74330 (CXCR3 antagonist) influence on chemokines under neuropathic pain” declare that the research was conducted in the absence of any commercial or financial relationships that could be construed as a potential conflict of interest.
